# Ehrlichiosis in a Recent Liver Transplant Recipient Leading to Multiorgan Failure

**DOI:** 10.1155/2022/3062836

**Published:** 2022-06-10

**Authors:** Fawwaz Almajali, Catherine Oleary, Taylor Hallcox, Justin Lok, Daniela Hermelin, Alexis Guenette, Mustafa Nazzal

**Affiliations:** ^1^Department of Surgery, Saint Louis University, St. Louis, MO, USA; ^2^School of Medicine, Saint Louis University, St. Louis, MO, USA; ^3^Department of Pathology, Saint Louis University, St. Louis, MO, USA; ^4^Department of Infectious Disease, Saint Louis University, St. Louis, MO, USA; ^5^Center for Abdominal Transplantation, Saint Louis University, St. Louis, MO, USA

## Abstract

Ehrlichia infection has a broad spectrum of diseases ranging from asymptomatic to fatal. While Ehrlichia often presents as a mild form of the disease in immunocompetent patients, immunosuppressed patients are at increased risk for a more virulent and potentially fatal infection. Our liver transplant patient presented with fever, persistent headaches, and negative Ehrlichia antibodies. Empiric antibiotic therapy was started and along with knowledge of prior tick infection, doxycycline was added. Subsequent positive PCR and observation of Ehrlichia chaffeensis in peripheral blood smear confirmed the diagnosis. The patient did recover from infection but not before it manifested in hepatic, renal, and pulmonary involvement. Therefore, a high level of suspicion is necessary for early detection and treatment initiation to prevent a devastating progression of the disease in immunosuppressed patients.

## 1. Introduction

Ehrlichiosis is used to classify diseases caused by a bacterial infection from either Ehrlichia chaffeensis, E. ewingii, or E. muris [1]. The disease is the most prevalent throughout the central and southern United States. Transmission primarily occurs via infected tick bites, with lone star tick being the most common vector [1] and white-tailed deer being the principal reservoir. The pathogens primarily infect leukocytes and subsequently circulate throughout the blood. Reports of Ehrlichia transmission have also been documented during blood transfusion, leuko-reduced red cells, platelets, and organ transplantation. Patients typically present with mild symptoms after a short incubation period of 1-2 weeks [1]. Clinical manifestations include fever, chills, headache, myalgias, nausea, vomiting, and diarrhea. Ehrlichia infections commonly go unnoticed during the initial stages due to the mild, nonspecific nature of symptoms. However, if antibiotic treatment is not initiated during the early stages, more severe symptoms will manifest. These may include hepatosplenomegaly, abdominal pain [2], respiratory failure, uncontrolled bleeding, multisystem organ failure, or death [1, 3].

Liver transplant patients are at a higher risk for a more virulent course given their immunosuppressed status. Numerous cases of ehrlichiosis in liver transplant patients have been previously reported. In addition, past cases have shown trends of liver failure following ehrlichiosis infection, including progressive transaminitis, hyperbilirubinemia, and hepatosplenomegaly [2]. In this case, we will discuss the clinical course and complications in the acute management of ehrlichiosis in a liver transplant recipient. We describe the clinical features of the disease, diagnostic techniques, and clinical response to interventions. Awareness and early recognition and treatment of ehrlichiosis in liver transplant recipients are critical in preventing liver failure.

## 2. Case Report

A 61-year-old female with a past medical history of nonalcoholic fatty liver cirrhosis with hepatocellular carcinoma who was 11 months postorthotopic liver transplant presented to an outside facility reporting three days of fevers associated with two weeks of dull, nonfocal headaches. The patient reported a left buttock tick bite one week prior. She was started on prophylactic meropenem, but was not tested for tick-borne illnesses, and was transferred for a higher level of care. On arrival at our facility, the patient complained of a persistent headache and was febrile to 102.9 degrees Fahrenheit and tachycardic with a heart rate of 110 beats per minute. Labs upon admission showed WBC 1.3, platelets 36, alkaline phosphatase 158 U/L, ALT 91 U/L, AST 198 U/L, total bilirubin 0.8 mg/dL; tacrolimus level 3.3, and INR 1.3. Other infectious workups including CMV, EBV, Lyme, Rocky Mountain spotted fever, Cryptococcus, and chlamydia were all negative. The patient's transplant immunosuppression medications, tacrolimus and myfortic, were held. She was then started on broad-spectrum antibiotics including cefepime, vancomycin, and doxycycline. CSF showed no evidence of infection. Cefepime and vancomycin were discontinued as blood cultures demonstrated no growth.

**Figure 1:**
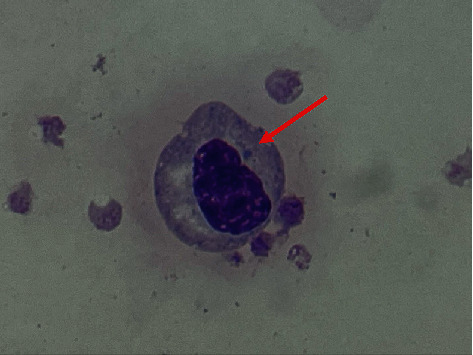
Peripheral blood smear reveals morulae (arrow) in the cytoplasm of a white blood cell.

Liver biopsy was obtained due to up trending liver enzymes, bilirubin, and INR (peak ALT 6448 U/L, peak AST 17601 U/L, INR 3.5, total bilirubin 6 mg/dL). It revealed evidence of diffuse necrosis though no suggestion of rejection. Peripheral blood smear was consistent with ehrlichiosis (Figure 1), despite Ehrlichia IgM/IgG being negative upon initial infectious workup, likely a false negative result secondary to immunosuppression. This was further confirmed with Ehrlichia chaffeensis PCR. Patient's therapy was deescalated to doxycycline for 2 weeks.

The following day, the patient displayed progressively increased work of breathing and oxygen requirement on high flow nasal cannula requiring intubation. Chest CT showed worsening bilateral lung opacities, for which cefepime and metronidazole were started. Pulmonary infectious workup was unrevealing. She was extubated after 4 days, and her respiratory status remained stable. She was started on dexamethasone due to secondary hemophagocytic lymphohistiocytosis diagnosis.

Due to increased encephalopathy, hypercapnia, and hyperkalemia, the patient was intubated again, and hemodialysis was initiated. A liver ultrasound showed a 3 cm pseudoaneurysm in the right lobe. A triple-phase CT showed a 2.8 × 2.6 cm hepatic segment 5/6 pseudoaneurysm (deemed high risk to intervene), large-volume ascites, and large right and small left pleural effusions with diffuse ground-glass opacities. Later that day, the patient developed PEA arrest, and ROSC was achieved after one round of CPR. Bronchoscopy showed mucus plugging throughout the trachea and bronchial airway.

After 3 days, the patient was extubated to nasal cannula, remaining hemodynamically stable off pressors. Liver allograft function remained stable, and the patient's mental status showed marked improvement. Renal ultrasound showed normal renal size with no abnormalities. The patient's bilirubin and alkaline phosphatase were noted to be trending upward indicating a cholestatic pattern, and MRCP showed no evidence of biliary stricture or defect. The patient continued to undergo intermittent hemodialysis and therapeutic large-volume paracentesis as needed for fluid overload and abdominal distension. Abdominal distension and AKI significantly improved over the hospital course. She was discharged home in stable condition and required temporary outpatient dialysis. Currently, she has normal liver function, and she is off hemodialysis with full renal recovery.

As illustrated in this case, early recognition and management of ehrlichiosis infection in the setting of the immunocompromised patient are imperative to quickly contain the infection and prevent detrimental complications of the disease.

## 3. Discussion

Ehrlichiosis has been described in several case reports as an etiology of infection in solid organ transplant recipients that most commonly occurs from the species Ehrlichia chaffeensis and results in human monocytic ehrlichiosis [4]. Ehrlichiosis infections predominantly occur in the South Central United States in the summer months [4]. Following a tick bite from the Lone Star tick (Amblyomma Americanism) and an incubation period of 1-2 weeks, most patients presented with fever and headaches, while a minority of presentations also included gastrointestinal symptoms [4, 5]. If the infection persists, multiorgan failure can manifest [4], including the kidney, liver, or heart failure. Due to their immunosuppressed status, transplant patients are more likely to develop a more severe illness. Notable laboratory findings that can aid in diagnosing ehrlichiosis include leukopenia, thrombocytopenia, and mild to moderate transaminitis; hyponatremia was infrequently reported.

Per CDC guidelines, a diagnosis of ehrlichiosis requires laboratory values to reflect antibody titers of at least 64 or a 4-fold change in antibody titers [6]. In an immunocompetent individual, IgG titers can appear as early as the first three weeks of illness. Initial antibody testing may appear negative in solid organ transplant recipients in the acute infection due to the patient's immunosuppressed status. PCR testing is the test of choice for acute ehrlichiosis infection due to its specificity (85%) and sensitivity (100%) [7]. In our case, we received diagnosis early due to the presence of the organism on peripheral blood smear as well as confirmation with a positive PCR.

Doxycycline is the agent of choice for treatment [4]. It has been shown that recovery time is significantly faster among hospitalized patients who received prompt initial treatment with doxycycline or chloramphenicol. Patients' average length of hospitalizations was decreased by 15 days with doxycycline and 11 days with chloramphenicol compared to patients initially treated with other antibiotics (*P* = .03) [8]. Further, a higher probability of severe illness/death has been reported in patients who did not receive tetracycline/chloramphenicol therapy until eight days or more following the onset of symptoms [8]..

As ehrlichiosis gains awareness, numerous cases have been reported in transplant patients. Thomas et al. (2007) reported 15 cases of ehrlichiosis in transplant patients, including seven kidneys, six hearts, one liver, and one lung transplants. All patients presented with leukocytopenia, fever, and thrombocytopenia. All patients were treated with doxycycline. The transplant patients did experience renal dysfunction and noted “the median baseline serum creatinine of the transplant patients was 1.4 mg/dL compared to 2.0 mg/dL at disease presentation (*P* = 0.0006)” [7]. Median laboratory values upon presentation for the transplant patients showed aspartate aminotransferase (AST) of 59 U/L, alanine aminotransferase (ALT) levels of 49 U/L, total bilirubin of 0.7 mg/dl, and alkaline phosphatase levels as 73 U/L [7]. The report described a mild to moderate disease course with successful treatment due to early doxycycline administration. PCR testing was also noted as a preferred method of testing, as antibodies are only present in the second to the third week of illness [7].

Cases of ehrlichiosis in transplant and nontransplant patients have resulted in organ failure, including liver dysfunction. For example, Schneider (2009) reported a 52-year-old man who presented with fever and chills. His serum creatinine, blood urea nitrogen (BUN) levels, and hepatic enzymes were elevated, and the patient suffered from acute renal and liver failure, respectively [9]. An ehrlichiosis infection was confirmed, and the patient was started on doxycycline and hemodialysis. Following treatment, the patient made a full recovery [9].

As ehrlichiosis infections become more prevalent, physicians need to be aware of its early presentations and more severe outcomes. In our case, we present a detailed account of a severe manifestation of an ehrlichiosis infection with multisystem organ failure due to delay in diagnosis as initial testing was negative, even after doxycycline treatment and required additional treatments, including hemodialysis and paracentesis. Our case strengthens the argument for early ehrlichiosis detection and administration of doxycycline.

## Data Availability

The data used to support the findings of this study are included within the article.
